# Characterization of Timed Changes in Hepatic Copper Concentrations, Methionine Metabolism, Gene Expression, and Global DNA Methylation in the Jackson Toxic Milk Mouse Model of Wilson Disease

**DOI:** 10.3390/ijms15058004

**Published:** 2014-05-07

**Authors:** Anh Le, Noreene M. Shibata, Samuel W. French, Kyoungmi Kim, Kusum K. Kharbanda, Mohammad S. Islam, Janine M. LaSalle, Charles H. Halsted, Carl L. Keen, Valentina Medici

**Affiliations:** 1 Department of Nutrition, University of California Davis, 3135 Meyer Hall, One Shields Avenue, Davis, CA 95616, USA; E-Mails: bichanhle@gmail.com (A.L.); clkeen@ucdavis.edu (C.L.K.); 2 Department of Internal Medicine, Division of Gastroenterology and Hepatology, University of California Davis, 4150 V Street, Suite 3500, Sacramento, CA 95817, USA; E-Mails: nshibata@ucdavis.edu (N.M.S.); chhalsted@ucdavis.edu (C.H.H.); 3 Department of Pathology, UCLA/Harbor Medical Center, 1000 West Carson Street, Torrance, CA 90502, USA; E-Mail: sfrench@labiomed.ord; 4 Department of Public Health Sciences, Division of Biostatistics, University of California Davis, One Shields Avenue, Med-Sci 1C, Davis, CA 95616, USA; E-Mail: kmkim@ucdavis.edu; 5 Research Service, Veterans Affairs Nebraska-Western Iowa Health Care System, VA Medical Center R-151, 4101 Woolworth Avenue, Omaha, NE 68105, USA; E-Mail: kkharbanda@unmc.edu; 6 Department of Medical Microbiology and Immunology, University of California Davis, One Shields Avenue, Tupper Hall, Davis, CA 95616, USA; E-Mail: saharul123@yahoo.com; 7 Department of Medical Microbiology and Immunology Genome Center, and MIND Institute, University of California Davis, One Shields Avenue, Tupper Hall, Davis, CA 95616, USA; E-Mail: jmlasalle@ucdavis.edu

**Keywords:** Wilson disease, copper, DNA, methylation, gene expression

## Abstract

**Background:**

Wilson disease (WD) is characterized by hepatic copper accumulation with progressive liver damage to cirrhosis. This study aimed to characterize the toxic milk mouse from The Jackson Laboratory (Bar Harbor, ME, USA) (tx-j) mouse model of WD according to changes over time in hepatic copper concentrations, methionine metabolism, global DNA methylation, and gene expression from gestational day 17 (fetal) to adulthood (28 weeks).

**Methods:**

Included liver histology and relevant biochemical analyses including hepatic copper quantification, *S*-adenosylmethionine (SAM) and *S*-adenosylhomocysteine (SAH) liver levels, qPCR for transcript levels of genes relevant to methionine metabolism and liver damage, and DNA dot blot for global DNA methylation.

**Results:**

Hepatic copper was lower in tx-j fetuses but higher in weanling (three weeks) and adult tx-j mice compared to controls. *S*-adenosylhomocysteinase transcript levels were significantly lower at all time points, except at three weeks, correlating negatively with copper levels and with consequent changes in the SAM:SAH methylation ratio and global DNA methylation.

**Conclusion:**

Compared to controls, methionine metabolism including *S*-adenosylhomocysteinase gene expression is persistently different in the tx-j mice with consequent alterations in global DNA methylation in more advanced stages of liver disease. The inhibitory effect of copper accumulation on *S*-adenosylhomocysteinase expression is associated with progressively abnormal methionine metabolism and decreased methylation capacity and DNA global methylation.

## 1. Introduction

Wilson disease (WD) is an autosomal recessive disorder characterized by mutations in the ATP7B gene which is responsible for copper (Cu) metabolism and excretion [1], with Cu accumulation in liver [2], and brain [3], that leads to progressive liver damage, as well as neurological and psychiatric manifestations [4,5]. The toxic milk mouse model of WD from The Jackson Laboratory (tx-j) has a G712D missense mutation in the second transmembrane region of ATP7B, which leads to hepatic Cu accumulation similar to the disease described in humans [6]. This Cu accumulation in hepatocytes results in microvesicular lipid droplets in association with damage to mitochondria [5], nuclei [7], and endoplasmic reticulum (ER) [8]. In addition, hepatic Cu accumulation correlates with down-regulation of gene transcripts related to lipid metabolism [9] including cholesterol synthesis [10].

Previous studies with the tx-j mouse model of WD have shown a progressive accumulation of liver Cu over 12 months, along with increases in hepatic apoptotic cells and hepatocyte metallothionein levels [11]. Apoptotic cell damage in the first six months of life was associated with increased levels of *Commd1*, a Cu binding protein, while decreased levels of proteins associated with inhibition of apoptosis were observed by eight months [4]. Reductions in liver Cu can be achieved in tx-j mice using the chelator tetrathiomolybdate [12].

We have previously demonstrated a close relationship between Cu accumulation and methionine metabolism in tx-j mice [9], as others have shown in other animal models of WD [13].

**Figure 1.**
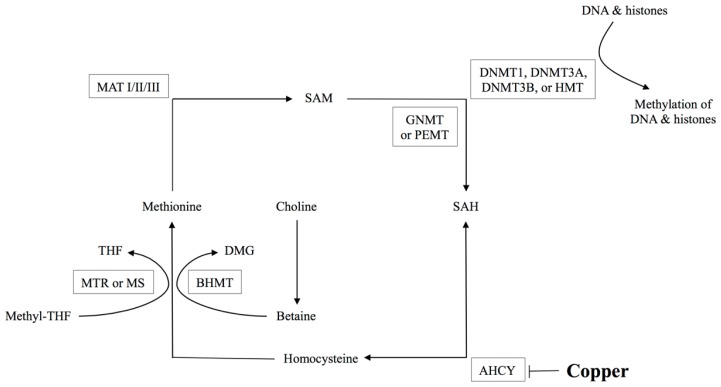
Methionine metabolism. S-adenosylmethionine (SAM) is the principal methyl donor for DNA and histone methylation reactions, whereas *S*-adenosylhomocysteine (SAH) inhibits all SAM-dependent methylation reactions. *S*-adenosylhomocysteinase (AHCY) is the bi-directional enzyme that hydrolyzes SAH to generate homocysteine. Methionine synthase (MTR or MS) remethylates homocysteine to form methionine that is in turn converted to SAM via the enzyme methionine adenosyltransferase (isoenzymes MATI/II/III). Homocysteine can also be remethylated to methionine via the betaine homocysteine methyltransferase (BHMT) catalyzed reaction that utilizes betaine. Dimethylglycine (DMG) is a byproduct of the BHMT catalyzed reaction and is also a potent inhibitor of BHMT. Tetrahydrofolate (THF); DNA methyltransferase (DNMT); histone methyltransferase (HMT); glycine *N*-methyltransferase (GNMT); phosphatidylethanolamine *N*-methyltransferase (PEMT); DNMTs, GNMT, and PEMT are major consumers of SAM in the liver [14].

As shown in Figure 1, methionine metabolism is essential for the production of methyl groups used in transmethylation reactions.

Methionine is an essential amino acid that must be ingested to ensure adequate provisions. In addition, methionine is generated from homocysteine via methionine synthase (MS or methionine transferase reductase (MTR) and BHMT-catalyzed reactions and is converted into *S*-adenosylmethionine (SAM), the main methyl donor for transmethylation reactions. DNA methyltransferases (DNMTs) catalyze DNA methylation reactions with production of *S*-adenosylhomocysteine (SAH). The maintenance of stable and balanced levels of SAM and SAH is crucial for cell physiology, as SAM is the main substrate for methylation reactions [15], whereas SAH is the major inhibitor for the same reactions. The ratio of SAM:SAH may be considered a relative index of methylation capacity [16], which in normal mouse liver tissue has been previously described to be in the range of 3 to 4 [14]. SAH regenerates homocysteine through *S*-adenosylhomocysteinase (AHCY), a bidirectional enzyme that favors the generation of SAH if the products homocysteine and adenosine are not removed. AHCY can be inhibited by Cu accumulation that results in elevated levels of SAH [9,17,18]. We recently associated hepatic Cu accumulation with reduced *Ahcy* gene expression and activity, subsequent elevations of SAH levels, reductions in the SAM: SAH ratio, and reduced DNA methylation. The above were associated with altered expression of genes relevant to liver injury [9]. This study also demonstrated that transcript levels of select genes related to methionine metabolism are down-regulated in tx-j mice and respond to choline supplementation with restoration of gene transcripts to control levels in fetal livers [19]. Others have shown that methionine metabolism and the need for methyl groups change over time, with the requirement for methyl groups being highest during gestational life [20].

In order to study the progression of WD, the present study examined the development of liver histology, methionine metabolism, transcript levels of selected genes central to lipid and methionine metabolism and global DNA methylation levels in tx-j mice from gestational day 17 (fetal) to postnatal week 28 (28 weeks). The study provides new temporal insights into relationships between hepatic Cu accumulation and liver damage, altered methionine metabolism, hepatic global DNA methylation, and regulation of gene expression.

## 2. Results and Discussion

### 2.1. Body and Liver Weights, Copper and Iron Status, SAM and SAH Levels, and Liver Histology

As shown in Table 1, body weights of the tx-j mice were significantly lower at three through 28 weeks of age than those of controls. Liver weights in the tx-j mice at three and 12 weeks of age were lower than those of controls. There was a significant increase in the liver/body weight ratio in the tx-j group at 20 and 28 weeks of age that coincided with a worsening of liver histology.

Liver Cu concentrations in fetal tx-j mice were significantly lower than in controls but by three weeks postnatal and thereafter, values were 2–50 times higher than in controls. Liver Cu concentration peaked at 20 weeks of age with significant age and genotype interaction (both *p* < 0.0001) indicating that liver Cu concentrations differed significantly over time between the tx-j and control mice. Liver iron concentrations in tx-j mice were similar to control levels at most time points, although values in the tx-j mice were 1.2 times higher than in controls at 28 weeks.

**Figure 2.**
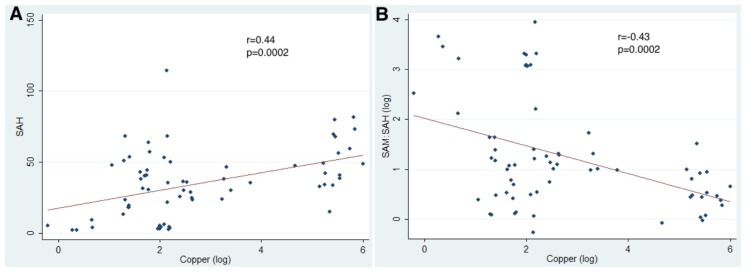
(**A**) Correlation between hepatic Cu concentration and SAH levels; (**B**) Correlation between hepatic Cu concentration and SAM:SAH levels (using data from all time points).

Postnatal hepatic SAM levels were higher in tx-j mice than in controls at three weeks with a significant interaction of age with genotype (*p* = 0.01) when examined over all time points. Liver SAH levels were similar in both groups at all time points except at three and 12 weeks with an overall significant interaction between age and genotype (*p* < 0.05) over time. The ratio of SAM to SAH (SAM:SAH ratio) was significantly lower at 12 weeks in the tx-j mice compared to ratios in the controls. When data from all time points were pooled, hepatic Cu concentrations were positively correlated with SAH levels (*r* = 0.44; *p* = 0.0002) and negatively correlated with SAM:SAH methylation ratio (*r* = −0.43; *p* = 0.0002) (Figure 2). These findings are compatible with the known inhibitory effect of Cu on *Ahcy* expression and enzymatic activity [9] with a predictive decrease in methylation capacity.

**Figure 3.**
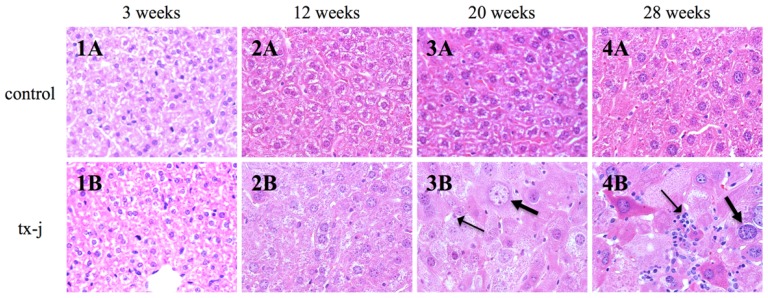
Histology images of tx-j and control livers from three to 28 weeks of age. All hematoxylin and eosin stained, 436×. Whereas liver histology was normal in tx-j mice at both three and 12 weeks of age (**1B** and **2B**), there was an increase in inflammatory infiltrates at 20 and 28 weeks (**3B** and **4B**, thin arrows), in association with giant nuclei and markedly increased cell size of the hepatocytes (**3B** and **4B**, thick arrows). Note that in tx-j mice hepatocyte cell diameters are 2.5 times and nuclear diameters are about two times larger than control mice [19]. Control mice had normal liver histology at all time points (**1A**, **2A**, **3A** and **4A**).

Although there were no changes at 12 weeks, we observed an increase in lymphocyte and PMN infiltration and in hepatocyte size in the tx-j group at 20 weeks. At 28 weeks, all the tx-j mice had increased hepatocyte sizes with giant nuclei, necrosis, increased lymphocytes, and PMNs. There were no histological changes in the control mice at all time points (Figure 3).

### 2.2. Transcript Levels of Selected Genes Related to Methionine and Lipid Metabolism

**Figure 4.**
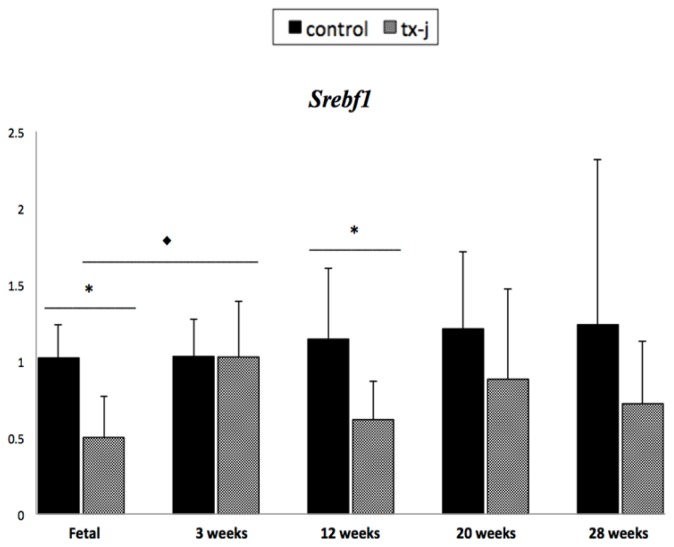

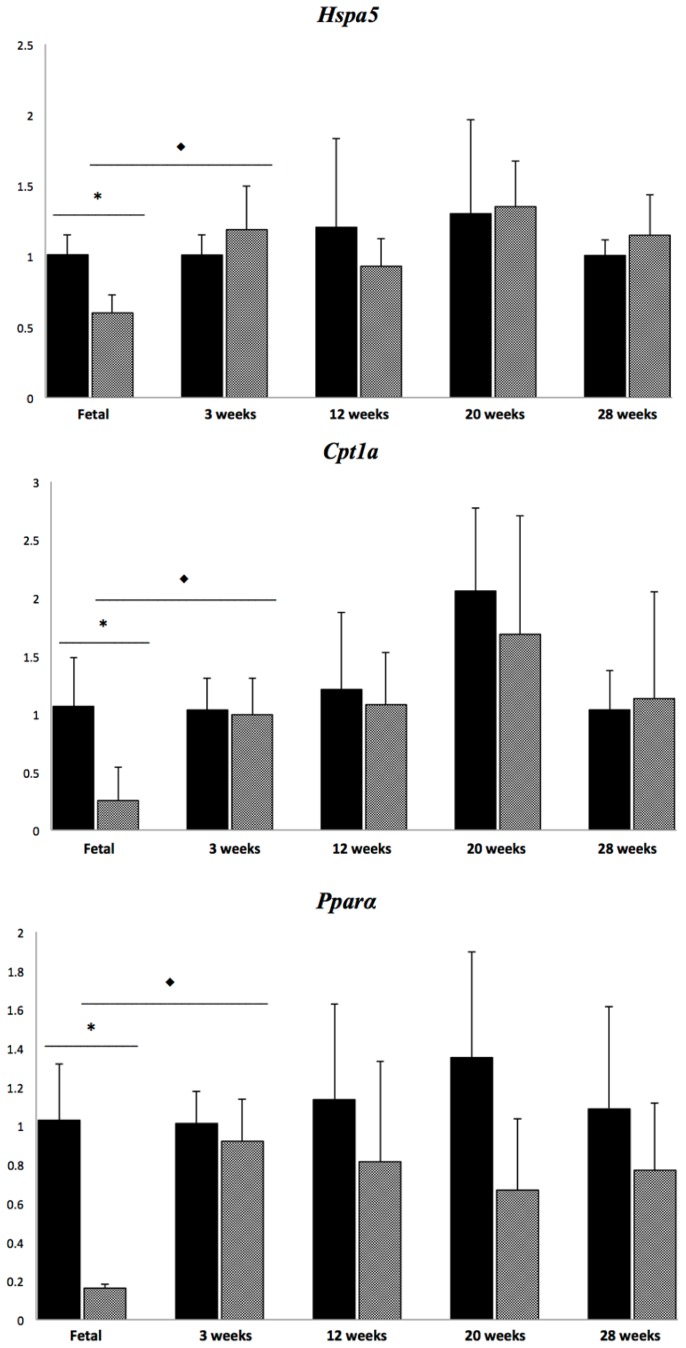

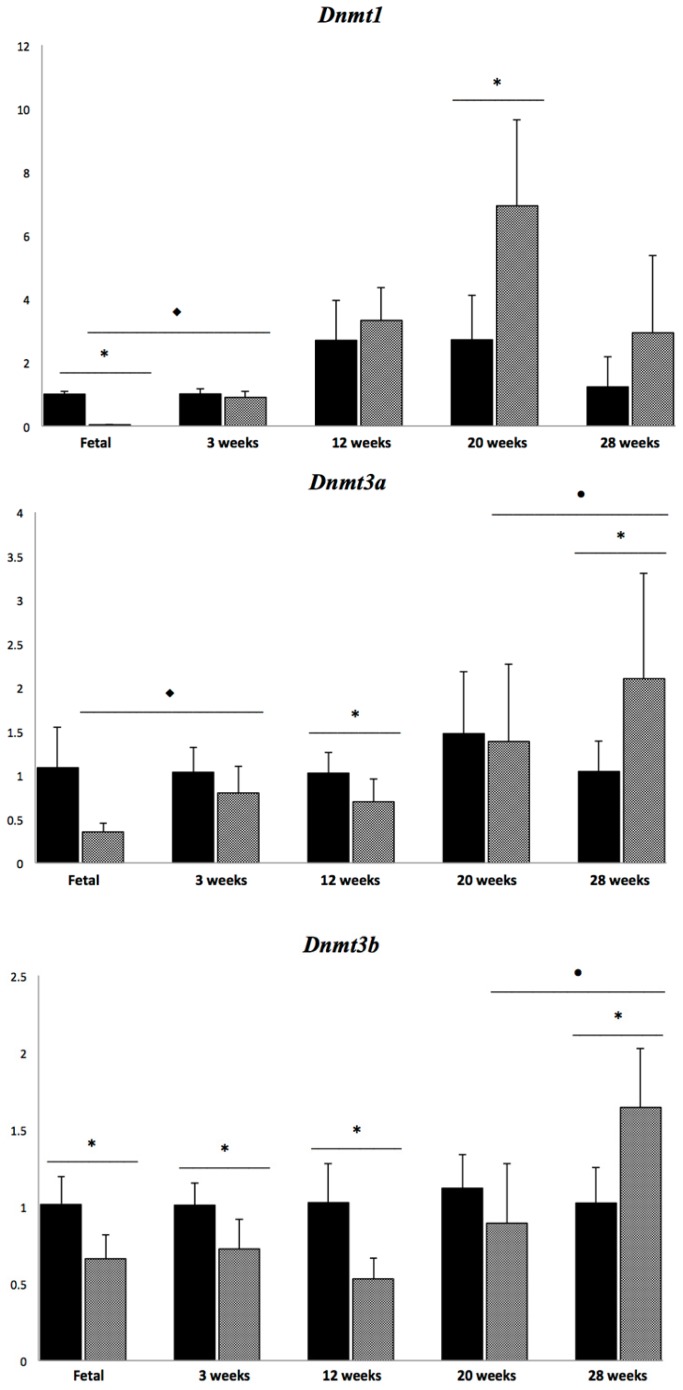

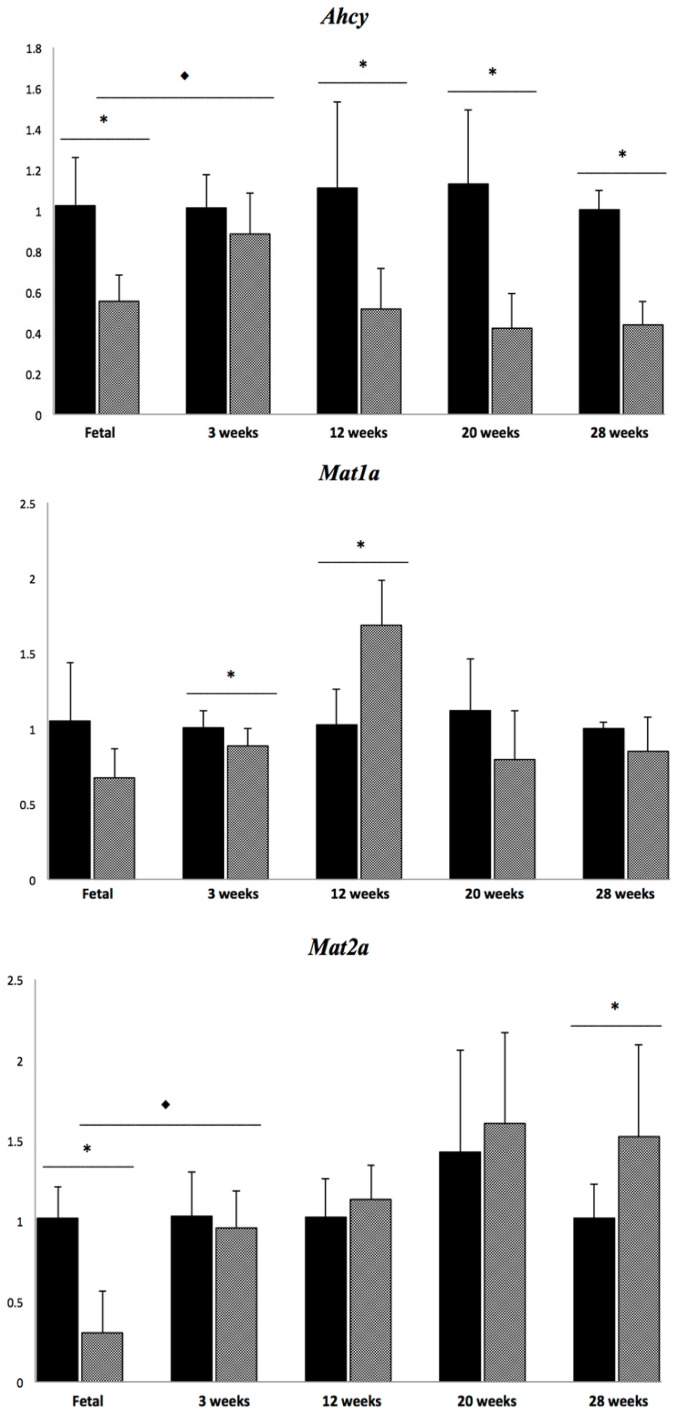

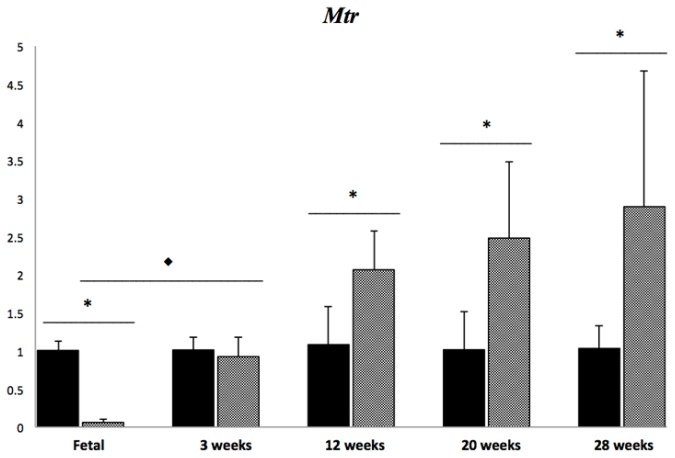
Transcript levels of genes related to lipid and methionine metabolism. *x*-axis: time points; *y*-axis: mean ± SD of relative expression of target genes normalized to *Gapdh*. Values with * are significantly different (*p* < 0.05) between groups within each time point. Values with ♦ are significantly different (*p* < 0.05) within the same genotype between fetal and three weeks. Values with ● are significantly different (*p* < 0.05) within the same genotype between 20 and 28 weeks. Genes include *Srebf1* (sterol regulatory element-binding protein) for lipogenesis, *Hspa5* (heat shock protein 5) for ER stress, *Cpt1A* (carnitine palmitoyl transferase 1A) and *Ppar*α (peroxisome proliferator-activated receptor α) for fatty acid oxidation, *Dnmt1* (DNA methyltransferase 1), *Dnmt3a* (DNA methyltransferase 3a), *Dnmt3b* (DNA methyltransferase 3b), *Ahcy* (*S*-adenosylhomocysteinase), *Mat1a* (methionine adenosyltransferase 1a), *Mat2a* (methionine adenosyltransferase 2a), and *Mtr* (methionine transferase reductase or methionine synthase) for methionine metabolism and DNA methylation. Fetal and three weeks data were previously published [19].

Transcript levels of 11 different genes were quantified including genes central to lipogenesis, fatty acid oxidation, methionine metabolism, and DNA methylation (Figure 4).

As previously reported [19], most of the fetal liver genes that were studied were down-regulated in the tx-j in comparison to control mice, with the exceptions of *Dnmt3a* and *Mat1a*. Subsequently, when comparing differences between gene transcript levels in control and tx-j mice at different time points, *Ahcy* was persistently down-regulated in tx-j mice except at three weeks, while *Srebf1* and *Dnmt3a* were down-regulated in tx-j mice at 12 weeks. *Dnmt1* was up-regulated at 20 weeks and *Dnmt3b* was down-regulated at three and 12 weeks. Both *Dnmt3a* and *Dnmt3b* were up-regulated in tx-j mice at 28 weeks. *Mat1a* was down-regulated at three weeks but was up-regulated at 12 weeks, and *Mat2a* was up-regulated in tx-j mice at 28 weeks. *Mtr* was up-regulated in tx-j mice at 12, 20, and 28 weeks. When comparing transcript levels in tx-j mice at different ages, transcript levels of almost all genes (except for *Dnmt3b* and *Mat1a*) were up-regulated at three weeks compared to fetal livers, whereas *Dnmt3a* and *Dnmt3b* levels were up-regulated at 28 weeks compared to 20 weeks. Consistent with our proposed interaction of Cu with methionine metabolism while considering all time points, *Ahcy* transcript levels were negatively correlated with hepatic Cu concentration (*r* = −0.58; *p* < 0.0001), and hepatic Cu was positively correlated with the expressions of *Dnmt1* and *Dnmt3a* (*r* = 0.68, *p* < 0.0001 and *r* = 0.28, *p* = 0.01, respectively).

### 2.3. Hepatic Global DNA Methylation

**Figure 5.**
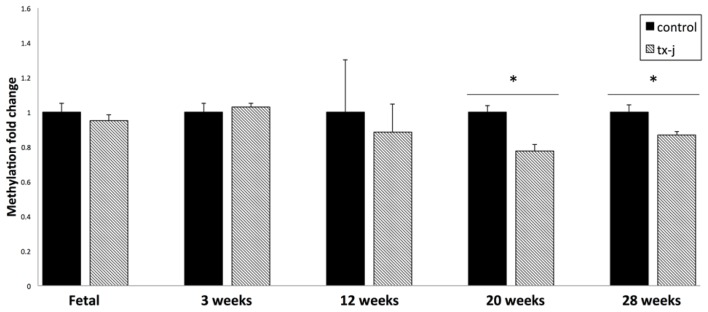
Hepatic global DNA methylation. Values with * are significantly different (*p* < 0.05) between tx-j *vs*. control within each time point. Values are expressed as fold change ± SD. Fetal and three weeks data were previously published [19].

DNA dot blots for global DNA methylation showed that there were no group differences in DNA methylation in fetal, three weeks, or 12 weeks old livers between tx-j and control mice. Starting at 20 weeks of age, tx-j mice DNA showed global hypomethylation compared to control mice with a significant interaction with age (*p* < 0.001), and this pattern persisted at 28 weeks (Figure 5). Pooling all time points, global DNA methylation levels were negatively correlated with *Dnmt3a* and *Dnmt3b* transcript levels (*r* = −0.26, *p* = 0.26; *r* = −0.27, *p* = 0.01) and were positively correlated with *Mat1a* transcript levels (*r* = 0.46, *p* < 0.001).

**Figure 6.**
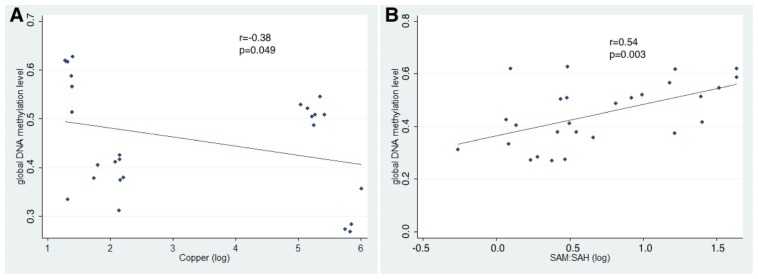
(**A**) Correlation between hepatic Cu concentration and global DNA methylation; (**B**) Correlation between SAM:SAH ratio and global DNA methylation (using data from 20 and 28 week old mice).

In addition, when using data only from 20 and 28 weeks old tx-j and control mice, global DNA methylation was negatively correlated with Cu concentrations (*r* = −0.38; *p* = 0.049) and SAH levels (*r* = −0.69; *p* ≤ 0.0001) and positively correlated with SAM:SAH ratio (*r* = 0.54; *p* = 0.003) (Figure 6).

The tx-j mice represent a valid model of WD and offer the opportunity to study the relations between Cu accumulation and methionine metabolism and their consequences on DNA methylation and gene expression. The rationale underlying our study was to describe the temporal relations between changes in methionine metabolism, DNA methylation, and gene expression, given that epigenetic marks are very dynamic, subject to influences that start during the early development and continue throughout life as a consequence of continuous environmental changes. The major findings of our study include the following: first that hepatic Cu was significantly lower in fetal livers of tx-j than in controls but postnatal values increased significantly over time with a plateau starting at 20 weeks of age; in contrast there were only minor differences in liver Fe concentrations. The finding that liver Cu concentrations were lower in fetal tx-j than controls may be attributed to the fact that adult tx-j dams are characterized by low circulating ceruloplasmin levels, with a putative Cu transport protein [19]. The observation that liver Cu concentrations reached a plateau around five months of age has been described before in other murine models of hepatic Cu accumulation [21] and may be attributed to adaptation mechanisms to hepatic Cu accumulation [4]. Hepatic Fe accumulation, as shown at 28 weeks, has been described previously in the LEC rat model of WD [22] and it can be speculated that this Fe accumulation plays a role in liver damage progression. Kato *et al*. [22] demonstrated that Fe-deficient diet could even prevent the development of fulminant hepatitis in LEC rats. While the mechanisms of hepatic Fe accumulation have yet to be elucidated, it is reasonable to speculate that since ceruloplasmin can contribute to Fe transport through hepatocyte basolateral membranes, low levels of this protein in WD may contribute to hepatic Fe accumulation [23]. Second, whereas almost all measured gene transcript levels were down-regulated in the tx-j fetal livers relative to control values, low levels of *Ahcy* persisted throughout all other time points. As a consequence of *Ahcy* down-regulation, the SAM:SAH methylation ratio was lower at 12 weeks of age although not consistently at all time points. Similarly, both SAM and SAH did not change consistently over time as expected since SAM was increased only at three weeks and SAH was increased only at three and 12 weeks which may be related to increased *Mtr* and *Mat* transcript and activity levels at this time points. A previous study reported transcript levels of enzymes related to methionine metabolism and mean SAM levels of 112.8 ± 12.4 nmol/g, SAH levels of 25.5 ± 3.9 nmol/g and their ratio of 4.4 ± 0.8 in mouse liver tissue [14]. Even though it is difficult to compare different mouse strains and animal ages, our SAM and SAH data in fetal livers are comparable with a previous report indicating very low SAH levels in fetal livers [24]. However, our present postnatal data are similar to the previous report in tx-j and control mice only at three weeks of age, whereas at later time points our SAM:SAH ratio is lower (range 1.8–2) due to progressive reduction of SAM levels relative to progressive increase of SAH levels. In an unrelated study, untreated C57BL/6J mice had similar SAM:SAH ratio of 2 ± 0.6 at five months of age [25]. The effects of Cu levels on methionine metabolism are also emphasized by the positive correlation between Cu and SAH levels, and negative correlation between Cu with *Ahcy* transcript levels and the SAM:SAH ratio. Two previous studies in toxic milk mice showed that *Ahcy* has Cu binding properties and its hepatic transcript levels can be reduced up to 42% in association with Cu accumulation [17,26]. Interestingly, Bethin *et al*. showed that Cu deficiency was also associated with 45% reduction of *Ahcy* hepatic levels [26], a finding that is similar to our results in fetal tx-j livers and that indicates that Cu metabolism must be strictly regulated to ensure stable *Ahcy* levels. Others showed that *Ahcy* deficiency in a 26-year-old man was associated with myopathy and developmental delay. Electron microscopy of the liver biopsy demonstrated extensive cytoplasmic lipid droplets [27]. Mutation of *Ahcy* in zebrafish was associated with SAH accumulation, increased levels of TNFα, and hepatic steatosis [28]. Third, although gene transcript levels were all down-regulated in fetal livers of tx-j mice, their improvement to control values after cross-fostering with control dams in all but *Dnmt3b*, *Ahcy*, and *Mat1a* at three weeks [19] suggests that maternal milk components may regulate hepatic gene expression to control levels. Of note, *Ahcy* transcript levels were reduced as well in fetal livers, similarly to all the other studied genes, despite the fact there was no Cu accumulation. We previously hypothesized that fetal hepatocytes in tx-j mice present dysregulation of cell cycle that has consequences on gene transcript levels, including *Ahcy*, and is corrected by methyl groups provision [19]. Delgado *et al*. [13] previously conducted a study on nine-week-old LEC rat models of WD, and showed down-regulation of *Ahcy* transcript levels. Interestingly, their finding of down-regulation of *Mtr* is opposite to present data and they failed to demonstrate any difference in SAM or SAH levels compared to wild type rats. These data suggest that various other factors may affect methionine metabolites levels.

Although the gene transcript levels were quite variable over time, global DNA hypomethylation was observed at 20 weeks of age and DNA methylation levels correlated negatively with *Dnmt3a* and *Dnmt3b* and positively with *Mat1a* transcript levels over all time points. The positive correlation between SAM:SAH and global DNA methylation supports the concept that Cu concentrations, methionine metabolism, and global DNA methylation can be tightly interrelated with gene expression regulation. We confirmed that *Ahcy* transcript levels were the most affected with consequences on SAM:SAH ratio and global DNA hypomethylation at 20 and 28 weeks. In addition, noteworthy, hepatic global DNA hypomethylation observed at the later time points was associated with more advanced inflammatory infiltrate. The finding of an association of decreased global DNA methylation at later time points with inflammatory infiltrates supports our previous results that chronic inflammation in WD is associated with an increased demand for methyl groups and consequent global DNA hypomethylation [9]. Previous reports suggested that progressive liver disease with early indication of fibrosis can be associated with changes in *Dnmt* levels and global DNA methylation in a mouse model of fibrosis induced by carbon tetrachloride [29]. Another study on human liver biopsies from patients with various degrees of severity of liver disease from chronic hepatitis to cirrhosis and hepatocellular carcinoma showed a progressive increase transcript levels of *Dnmts* in association with more advanced liver disease [30], an observation that is similar to our current study. In addition, previous reports described increased *Dnmts* transcript levels correlating with reduced global DNA methylation likely as a result of a compensatory mechanism [31,32]. The design of our present study that is based on timed changes in methionine metabolism and DNA methylation does not allow us to determine if DNA methylation is the cause of worsening liver pathology or if it is epiphenomenon or a consequence of it. However, it is possible that both hypotheses may be true. As shown by our data, the changes in methionine metabolism including down-regulation of *Ahcy* expression in the fetal liver preceded Cu accumulation and inflammation and amplified liver damage, which in turn could increase a requirement for methyl groups. Global DNA hypomethylation has consequences on the regulation of gene expression as shown in a recent study of progressive liver fibrosis [29]. In addition, improvement of global DNA methylation after folate supplementation was associated with decreased inflammation in gastric mucosa infected by Helicobacter Pylori [33]. To summarize, our results indicate that the tx-j mouse model of WD is characterized by changes in methionine metabolism that are evident from the gestational phase of development onwards. *Ahcy* down-regulation is persistent over time and is negatively correlated with increasing hepatic Cu concentration, a finding that is consistent with the known inhibitory effects of Cu on its expression [9,26]. As a consequence of *Ahcy* down-regulation, there is a reduction in liver SAM:SAH levels which correlated with global DNA methylation observed at the later time points.

## 3. Experimental Section

### 3.1. Animals and Care

The study was conducted using *C3HeB/FeJ-Atp7b^tx-J/J^* (tx-j) mice and *C3HeB/FeJ* (control) mice. All mice were bred in-house on the UC Davis campus (Davis, CA, USA). All animals had access to Purina LabDiet 5001 stock (13 μg Cu, 270 μg Fe, 70 μg Zn per g diet, 28% Kcal protein, 12% Kcal fat, and 60% Kcal carbohydrate) and deionized water *ad libitum*. Animals were group-housed in polycarbonate cages and maintained according to guidelines set forth by the American Association for Accreditation of Laboratory Animal Care IACUC (Institutional Animal Care and Use Committee, UC Davis, Davis, CA, USA). The animal room was maintained at 20–23 °C and 45%–65% relative humidity with a 14 h light/10 h dark light cycle.

Dams were anesthetized at gestational day 17 (GD17) via CO_2_ anesthesia followed by cervical dislocation, and fetal livers were pooled and flash-frozen in liquid nitrogen (*n* = 8 pools of control fetal livers; *n* = 5 pools of tx-j fetal livers). All postnatal tx-j pups were cross-fostered to a lactating control dam between post-partum day 0 and 6 due to an insufficient amount of Cu to sustain neonatal development and growth in the milk of a tx-j mouse. Mice at 3 (control *n* = 11; tx-j *n* = 10), 12 (control *n* = 8; tx-j *n* = 7), 20 (control *n* = 9; tx-j *n* = 6), and 28 (control *n* = 7; tx-j *n* = 7) weeks of age were euthanized via isoflurane anesthetic followed by exsanguination and cervical dislocation. Sections of postnatal livers were placed in formalin or stored in the −80 °C freezer until analysis. Fetal livers and 3 weeks livers have been previously published [19]. The protocol was approved by UC Davis IACUC (protocol#16172, approved on 21 October 2010).

### 3.2. Hepatic SAM and SAH

Liver levels of SAM and SAH were measured through high-performance liquid chromatography. Liver tissue was homogenized in cold 0.5 N perchloric acid at a ratio of 50 mg tissue: 400 μL perchloric acid and subsequently centrifuged at 14,000 rpm for 10 min. The supernatant was then filtered through a 0.2 μm syringe filter, aliquoted, and stored in the −80 °C freezer until HPLC analysis could be performed to quantify SAM and SAH [34]. HPLC analysis was done within 4 weeks of tissue collection to ensure sample stability. This method has been confirmed independently in another laboratory [35].

### 3.3. Hepatic Copper and Iron

Approximately 100 mg of liver tissue was digested with concentrated nitric acid and then wet-ashed for analysis using flame atomic absorption spectroscopy [36].

### 3.4. Liver Histology

Liver tissue from both control and tx-j mice were prepared by staining sections with hematoxylin and eosin. Images were blind-evaluated for mitosis, nuclei, lymphocytes, PMNs, hepatocyte size, and fibrosis.

### 3.5. Transcript Levels of Selected Genes by qPCR

RNeasy Mini Kit (QIAGEN, Valencia, CA, USA) was used to isolate total RNA from liver tissue. Purity and concentration of extracted RNA was determined by NanoDrop spectrophotometry (Cole-Parmer, IL, USA) and RNA integrity determined by gel electrophoresis. Samples were stored at −80 °C until analysis. cDNA was synthesized using the SuperScript III First-Strand cDNA synthesis kit (Invitrogen, Carlsbad, CA, USA). SYBR green was used to detect transcript levels of 11 selected genes (Table 2); all samples were run in triplicate. Primers for cDNA sequences were designed using AB Tm calculator (http://www6.appliedbiosystems.com/support/techtools/calc/index.cfm), NCBI Primer-BLAST (http://www.ncbi.nlm.nih.gov/tools/primer-blast/), and Premier Biosoft International Beacon Designer (http://www.premierbiosoft.com/qOligo/Oligo.jsp?PID=1). Efficiency of all primers was >95% and specificity checked via melt curve and gel electrophoresis. All primers were used at a concentration of 300 nM except for *Mtr* primers which were used at a concentration of 900 nM. qPCR was done on the AB ViiA 7 Real-Time PCR System (Applied Biosystem, Foster City, CA, USA).

Reactions were run at 50 °C for 2 min and 95 °C for 10 min, then 40 cycles at 95 °C for 15 s and 60 °C for 1 min. All *C*q expression values were normalized to *Gapdh* and relative expression was calculated using the equation 2^−ΔΔ^*^C^*^q^, where ΔΔ*C*q = Δ*C*q (sample) − *C*q (calibrator).

### 3.6. Global DNA Methylation via Dot Blot Analyses

DNA was isolated from liver samples using the QIAGEN DNeasy Blood & Tissue Kit (Valencia, CA, USA). An established method was used to measure relative methylation against 5-methylcytosine [32]. 50 ng of genomic DNA was alkaline denatured and spotted on a nitrocellulose membrane. After UV cross-linking, membranes were blocked in LiCor Odyssey Blocking Buffer (LiCor Biosciences, Lincoln, NE, USA), and incubated with anti-5-methylcytosine (Eurogentec, Fremont, CA, USA) in Blocking Buffer + 0.1% Tween-20 overnight at 4 °C. Membranes were washed in 1× PBS + 0.1% Tween-20, followed by incubation with LiCor 700-IR secondary antibody (LiCor Biosciences, Lincoln, NE, USA). Blots were washed again and imaged using the Licor Odyssey Imager (LiCor Biosciences, Lincoln, NE, USA). Blots were rinsed with 2× SSC buffer then equilibrated in PerfectHyb Plus Hybridization Buffer (Sigma, St. Louis, MO, USA) at 42 °C. Blots were hybridized with heat-denatured biotin-labeled gDNA overnight at 42 °C, washed with a high stringency buffer, then blocked again with Blocking Buffer. Blots were further incubated with LiCor Streptavidin 800-IR secondary antibody (LiCor Biosciences, Lincoln, NE, USA) in Blocking Buffer + 0.1% Tween-20 for 1 h at room temperature, then imaged on the LiCor Odyssey Imager (LiCor Biosciences, Lincoln, NE, USA). LiCor Odyssey software (LiCor Biosciences, Lincoln, NE, USA) was used to analyze the integrated intensities. Methylation signal was normalized to the total DNA signal [9] and results expressed as fold change relative to the control group for each time point.

### 3.7. Statistical Analysis

Statistical analysis was performed using a two-way ANOVA with an interaction term. Where the overall ANOVA was significant, we identified genotypes (tx-j *vs*. control) and cross-sectional time points (fetal and 3, 12, 20, or 28 weeks of age) that differed significantly using Tukey’s multiple comparison procedure and maintained the family-wise error rate at 0.05. Pearson correlation coefficient and its *p*-value for significance of correlation were calculated to assess the magnitude and direction of an association between two given variables. For data that were highly skewed, we applied a natural log transformation to achieve normality prior to statistical analysis and significance testing was done on a log-transformed scale. All reported *p*-values are based on two-sided tests. A *p*-value <0.05 was considered significant. All statistical analyses were performed using SAS, Version 9.4 (SAS Institute, Cary, NC, USA).

## 4. Conclusions

In the present study, we observed that transcript levels of genes related to methionine metabolism are aberrant in the liver of the tx-j mouse model of WD from late gestation to adult life in parallel with increasing levels of hepatic Cu and abnormal histopathology. The accumulation of hepatic Cu correlated with decreasing expression of *Ahcy*, with consequent increases in SAH levels and reduction in SAM:SAH methylation ratios at several time points. In more advanced phases of liver disease, tx-j mice presented global DNA hypomethylation which in turn correlated with the SAM:SAH ratio. The interaction between Cu accumulation and methionine metabolism is a crucial mechanism of disease onset and progression in WD indicating that there is a close connection between genetic and epigenetic mechanisms that ultimately determinates the phenotypic expression of this condition.

## Figures and Tables

**Table 1.** Developmental changes (fetal–28 weeks) in body and liver weights, hepatic copper and iron, and SAM and SAH concentrations. Values are expressed as mean ± SD.

Analysis	Fetal		3 weeks		12 weeks		20 weeks		28 weeks	
					
	Control (*n* = 8)	Tx-j (*n* = 5)	Control (*n* = 11)	Tx-j (*n* = 10)	Control (*n* = 8)	Tx-j (*n* = 7)	Control (*n* = 9)	Tx-j (*n* = 6)	Control (*n* = 7)	Tx-j (*n* = 7)
Body weight (g)	ND	ND	9.29 ± 1.03	7.91 ± 0.84 *	32.3 ± 2.42	27.1 ± 1.45 *	34.8 ± 2.14	28.3 ± 1.10 *	38.7 ± 4.31	32.2 ± 2.58 *
Liver weight (g)	ND	ND	0.40 ± 0.060	0.35 ± 0.045 *	1.65 ± 0.12	1.40 ± 0.068 *	1.74 ± 0.14	1.72 ± 0.089	1.84 ± 0.27	1.68 ± 0.27
Liver/body ratio	ND	ND	0.043 ± 0.0035	0.044 ± 0.0024	0.051 ± 0.0017	0.052 ± 0.0024	0.050 ± 0.0026	0.061 ± 0.0030 *	0.047 ± 0.0021	0.052 ± 0.0045 *
Copper (μg/g) **	7.83 ± 0.72	1.49 ± 0.48 *	12.17 ± 1.72	30.65 ± 7.73 *	5.17 ± 0.99	223.16 ± 52.8 *	7.44 ± 1.84	351 ± 40.5 *	3.86 ± 0.19	190 ± 23.4 *^,^**•**
Iron (μg/g) **	53.34 ± 7.73	48.94 ± 6.08	23.54 ± 3.39	26.16 ± 5.68	68.76 ± 11.54	79.52 ± 35.84	84.60 ± 13.27	91.66 ± 3.42	97.58 ± 14.92	124.07 ± 7.89 *^,^**•**
SAM (nmol/g)	105.19 ± 24.83	82.13 ± 14.52	86.97 ± 17.82	122.55 ± 50.37 *	84.9 ± 11.9	76.6 ± 26.3	82.7 ± 17.2	99.9 ± 10.6	74.4 ± 13.2	77.2 ± 7.91
SAH (nmol/g)	4.04 ± 1.21	4.67 ± 2.88	25.94 ± 8.86	36.55 ± 13.35 *	42.5 ± 10.4	57.2 ± 15.7 *	56.6 ± 26.3	67.7 ± 13.4	28.2 ± 16.8	34.5 ± 11.4
SAM/SAH ratio	27.59 ± 10.16	23.17 ± 12.77	3.89 ± 1.84	3.39 ± 0.89	2.14 ± 0.69	1.41 ± 0.59 *	1.82 ± 1.13	1.51 ± 0.26	3.76 ± 1.33	2.12 ± 0.52

Values with * are significantly different (*p* < 0.05) between tx-j *vs*. control at each time point; ** Copper and iron concentrations at the three weeks time points are from livers of 8 controls and 5 tx-j mice; Values with • are significantly different (*p* < 0.05) within the same genotype between 20 and 28 weeks. ND = not determined. Fetal and three weeks data were previously published [19].

**Table 2.** qPCR primer sequences of selected genes.

Gene	Sequence 5′ to 3′	Accession	Position (bp)	Exon-exon overlap	% Primer efficiency
*Srebf1*-F	CTGGCTTGGTGATGCTATGTTG	NM_011480.3	3901–3922	No	104.9
*Srebf1*-R	GACCATCAAGGCCCCTCAA		3978–3960	No	

*Hspa5*-F	GTGGAGATCATAGCCAACG	NM 022310.3	383–401	No	102.9
*Hspa5*-R	CACATACGACGGCGTGATGC		433–414	No	

*Cpt1a*-F	GGAGGAGACAGACACCATCCA	NM 013495.2	1044–1064	Yes	104.1
*Cpt1a*-R	CGTCATGGTAGAGCCAGACCTT		1138–1117	No	

*Pparα*-F	CGATGCTGTCCTCCTTGATGA	NM_011144.6	1401–1421	No	98.5
*Pparα*-R	GAAGTCAAACTTGGGTTCCATGAT		1528–1505	No	

*Dnmt1*-F	CCAGCTGCCAAACGGAGA	NM_001199431.1	1060–1077	Yes	102
*Dnmt1*-R	CCTCGGGAGTCTCTGGAGCTA		1123–1103	No	

*Dnmt3a*-F	CACTGGAGTAGGCGCTGAGAC	NM_007872.4	7635–7655	No	101.6
*Dnmt3a*-R	CAGCAAAGGGCCTTCCATAG		7699–7680	No	

*Dnmt3b*-F	CCGTTCGACTTGGTGATTGG	NM_001003961.4	2352–2371	No	101.3
*Dnmt3b*-R	GGGCAGGATTGACGTTAGAGAG		2412–2391	No	

*Ahcy*-F	ATCCTTGGCCGGCACTTT	NM_016661.3	949–966	Yes	97.1
*Ahcy*-R	TTCTTTAGCCAGTAGCGGTCCA		1100–1079	No	

*Mat1a*-F	TCTGTCCCATACTCACCTCTTCAG	NM_133653	1683–1706	No	98.4
*Mat1a*-R	TGCCCTGAGGGTAGAAGGC		1771–1753	No	

*Mat2a*-F	CAGGAGACCAGGGTTTGATGTT	NM_145569	517–538	Yes	95.8
*Mat2a*-R	GCGTAACCAAGGCAATGTACC		653–633	No	

*Mtr*-F	CTGCAGATGTGGCCAGAAAAG	NM_001081128	580–600	Yes	98.9
*Mtr*-R	CAGCCACAAACCTCTTGACTCC		648–627	No	
